# Perspectives on pharmacologic strategies in the management of meningoencephalomyelitis of unknown origin in dogs

**DOI:** 10.3389/fvets.2023.1167002

**Published:** 2023-05-10

**Authors:** Michaela J. Beasley, Andy Shores

**Affiliations:** College of Veterinary Medicine, Mississippi State University, Starkville, MS, United States

**Keywords:** immunosuppression – general, cytarabine (Ara-C or cytosine arabinoside), Cytosar, meningoencephalitis, cyclosporine (CyA)

## Abstract

There are many non-infectious inflammatory diseases, assumed to be immune-mediated in origin, recognized to affect the nervous system in canine patients. Concentrating on meningoencephalomyelitis of unknown origin, we will discuss the medications used to treat the underlying disease process, focusing on their adverse effects, therapeutic monitoring when necessary and effectiveness. The literature overwhelmingly supports the use of a steroid/ Cytosar^®^ or steroid/ cyclosporine treatment protocol with the steroid tapered after the acute phase of the disease, leaving the secondary medication to control the disease long term. The decision on when and how quickly to taper the steroid is clinician dependent as a best practices has not been established in the literature. Also discussed will be the supportive care treatments often needed in the acute phase of these patients’ diagnosis and treatment such as anti-edema and anti-epileptic agents.

## Introduction

Chemotherapies and other immune modulating medications are often used in veterinary neurology to treat various immune-mediated (noninfectious inflammatory) diseases. These diseases most commonly include meningoencephalomyelitis of unknown origin (MUO), steroid responsive meningitis arteritis, idiopathic tremor syndrome, eosinophilic meningoencephalitis, and inflammatory neuropathies and myopathies (1–5). Herein we will briefly discuss the diagnosis of, followed extensively by the treatment, specifically, of MUO.

### Diagnosis

Meningoencephalomyelitis of Unknown Origin (MUO) is a term used to describe an inflammatory disease of the central nervous system of dogs and, as the name implies, an etiology has not been identified. Histopathology further distinguishes it as granulomatous meningoencephalitis (GME) or necrotizing encephalitis (NE). Necrotizing encephalitis is further sub-characterized as necrotizing leuko- or meningo- encephalitis. Granulomatous meningoencephalitits is the only of the three known to affect the spinal cord (1–5).

MUO should be considered based on signalment and presenting clinical signs. Most dogs will fall into the small to medium size (<10kgs), young adult (3–7yo) categories with GME typically being older (4–8yo) than their NE counterparts (<4yo) (1–5). The following breeds are considered predilected: Toy Poodle, Pug, Maltese, Yorkshire Terrier, Chihuahua, French Bulldog, Papillion, Shih Tzu, Pekinese, West Highland White Terrier, Coton de Tulear, and Brussels Griffon (2). Dogs with MUO tend to have a subacute to acute onset of multifocal forebrain and/or brainstem signs, although GME can present with a strictly spinal or ocular localization (1–5). Large breed and older dogs, although less common, have been diagnosed with MUO (2, 6).

Definitive antemortem diagnosis requires referral for magnetic resonance imaging (MRI) and cerebrospinal fluid (CSF) analysis. Typical MRI findings include multifocal CNS lesions that are irregular and ill-defined and that are hyperintense on T2-weighted and FLAIR images with variable contrast enhancement. In general, CSF changes include an elevated total protein and a mononuclear or mixed pleocytosis (1–5). Occasional dogs will have normal protein and cytology, reported in 3–57% (1–3), however, as reported and in our experience, these patients have already been on a corticosteroid regime (3), highlighting the importance of a complete diagnostic work up before initiating treatment (7). Often not considered in clinical presentations, but certainly a gold standard would be a cerebral biopsy to confirm a diagnosis (1, 5). In addition, patients should also be screened, via PCR or serology, for the common infectious diseases capable of causing meningoencephalitis in their geographical region. These can include tick borne, protozoal viral and fungal diseases (1–3, 5, 7).

### Treatment/discussion

While literature abounds on the topic of the diagnosis and treatment of MUO, guidance on specifics for treatment (which medications and exact dosages and weaning protocols) varies by author (8), therefore, the recommended treatment protocol outlined in his article represents literature guidance and where specified the authors’ opinions formed from years of clinical experience.

#### Supportive care for the MUO patient

Apart from the treatment of the underlying disease process, these patients may require anti-edema as well as anti-epileptic therapies (1).

Anti-edema medications such as mannitol and 3% hypertonic saline are administered based on severity of neurologic dysfunction, severity of intracranial edema seen on MRI or a declining neurologic status during serial examinations of the patient (1). Mannitol is an osmotic diuretic and obligate extracellular solute used to decrease intracranial pressure through reduction in CSF production and via osmotic pull of extracellular fluid into the intravascular space which also serves to reduce blood viscosity improving brain oxygen delivery. Mannitol (20–25%), kept in a fluid warmer/incubator to prevent crystallization, can be dosed at 0.5–1 g/kg intravenously administered through a filtered needle, over 15–30 min. Side effects of mannitol include volume expansion (as fluid shifts into the vascular space) followed by depletion (as the solute is filtered in the glomerulus), hyponatremia, and acute kidney injury especially when serum osmolality is raised greater than 320 mOsm/L (9, 10). To minimize side effects, measured (not calculated) serum osmolality and electrolytes should be monitored in patient receiving mannitol therapy, in the absence of this monitoring, it has been suggested to administer no more than 3 doses in a 24-h period (9). Hypertonic saline allows for similar osmotic effects as mannitol due to the inability for sodium to cross the blood brain barrier while offering volume expansion without the diuretic effects of mannitol (10). It is important to note the concentration of hypertonic saline being used to calculate dose. The 3% is dosed at 5.4 ml/kg intravenously while the 7% is recommended at 4 ml/kg administered over 2–5 min. The 23.4% requires dilution with an colloid agent before administration (1:2 dilution ratio dosed at 4 ml/kg) (10). Side effects of hypertonic saline are most commonly linked to the patients serum sodium levels which should be checked prior to hypertonic saline administration. Hyponatremic patients should not be administered hypertonic saline to avoid myelinolysis and patient receiving multiple doses of hypertonic saline should have their serum sodium monitored with the goal to not exceed 160 mEq/L (10). Patients receiving either anti-edema therapy should also be treated with isotonic fluid therapy and those patients at risk for fluid overload should be dosed using the low end of the dosage range for both mannitol and hypertonic saline (9, 10).

Due to lesions in the forebrain, patients with MUO may present due to seizure activity or develop it during treatment (1, 11). Breakthrough seizure activity can be emergently treated with intravenous benzodiazepine boluses and/or continuous rate infusions while maintenance anti-epileptic medications are chosen and implemented based on the preferences of the clinician (12). Diazepam (0.5–2 mg/kg) and midazolam (0.06–0.3 mg/kg) can be bolused intravenously, recommending to switch to a constant rate infusion (diazepam 0.1–0.5 mg/kg/hr or midazolam 0.1–0.5 mg/kg/hr) after three boluses have been required (12, 13). The authors’ preference is midazolam boluses at 0.3 mg/kg followed by a midazolam constant rate infusion of 0.3 mg/kg/hr. Commonly used maintenance therapies available in parental formulations include phenobarbital and levetiracetam (12). Phenobarbital is loaded with 15–20 mg/kg IV as a singular dose or divided into multiple doses administered within a 24 h period then continued as maintenance therapy at 2.5–3 mg/kg twice daily (14). Levetiracetam is loaded with a 60 mg/kg intravenous bolus, with the literature not in agreeance on whether or not to dilute and over what time to give the medication, 1–2 min vs. 15 min then continued at 20 mg/kg three times daily (15, 16). The authors choose to dilute levetiracetam 1:1 with 0.9% NaCl given as a slow IV push followed by maintenance dosing of 30 mg/kg three times daily. Between phenobarbital and levetiracetam, the authors prefer levetiracetam due to its low side effect profile, specifically the lack of mental state alteration (sedation), making it ideal when continuous serial neurologic examinations are to be performed in patients recently diagnosed with MUO to monitor for disease progression. As an additional benefit, as these patients improve and are discharged from the hospital, levetiracetam offers a convenient commercially available liquid formulation to fit the often-small size patients diagnosed with MUO and allows for easy adjustment of dose as the patients gain body mass while on immune-suppressant medications.

#### Initial treatment of the MUO patient

After initial diagnostics (supportive MRI and CSF analysis results) stable patients are treated with an anti-inflammatory dose of glucocorticoid while simultaneously testing for infectious diseases (1, 5). If using prednisone/ prednisolone the dose most often reported during this time is 0.25–1 mg/kg/day (1, 5). If there is a reasonable suspicion of infectious disease, it is appropriate to begin a course of doxycycline, clindamycin and/or fluconazole. Once confirmed negative for infectious diseases, the glucocorticoid dose is increased to an immunosuppressive level and a second immunosuppressive medication is initiated based on clinician preference. The addition of a second immunosuppressive medication may be due to severity of neurologic disease, neurologic deterioration despite glucocorticoid therapy and the want to minimize the effects of long term glucocorticoid therapy side effects (1). Common secondary medications include: cytarabine, cyclosporine, azathioprine, procarbazine, and mycophenolate mofetil. Lomustine has been reported, but its use did not result in a significantly longer survival time compared to prednisone alone (17). Factors takin into account include: ability or inability to frequently transport patient to hospital for medication monitoring, vicinity to a hospital capable of chemotherapy administration, monetary concerns, patient temperament, ease of catheterization and venipuncture and concurrent medical issues of the patient and owner.

In severely affected patients, due to the low yield of infectious testing, immunosuppressive treatment is initiated prior to serology results, following owner counseling on the risks of immunosuppression if an infectious disease is present (5).

#### Steroid therapy

The authors will maintain glucocorticoid treatment steady for the first 3 months after diagnosis, commonly using prednisone/ prednisolone at 1–2 mg/kg/day (18), saving 2 mg/kg twice daily dosing for those most affected patients and then only for the first few days to 1 week of treatment (19). In addition to their anti-inflammatory mechanism of action, steroids will also serve to reduce both vasogenic edema and cerebrospinal fluid production rate, further aiding in reduction of intracranial pressure. The prednisone serves as the primary suppressor of the immune-mediated component of the disease while allowing for time to onset of action for the secondary immunosuppressive medications. In the case of cyclosporine, titration of the medication based on whole blood levels is needed during this time.

The authors decision on 3 months is based on studies demonstrating higher risk of relapse if CSF is still abnormal at 3 months, or the treatment taper starts before resolution of MRI abnormalities (18). Ideally, repeat diagnostics (MRI and CSF analysis) would be performed after 3 months of treatment, however, this may not be financially feasible for owners (18). When repeat diagnostics cannot be performed, tapering is not started until 3 months post diagnosis or once maximal improvement of neurologic signs has been reached, whichever comes last.

The authors will decrease the steroid dose by 25% every 6–8 weeks eventually reducing the dose to 0.25–0.5 mg/kg once every 3 days. Published studies recommend tapering every 3–6 weeks (1, 5, 19, 20). Each recurrent dose taper is contingent on the lack of clinical sign recurrence, and some patients will not be able to reach a full wean off steroids. Most owners will notice the common side effects of polyphagia, polydipsia, and polyuria, but find the polyuria the most frustrating aspect. In those households where the polyuria is too exacerbating, the use of desmopressin (5mcg/dog administered subcutaneously twice daily) can ameliorate those clinical signs, but it is necessary to watch for hyponatremia in these patients (21).

Studies have found single agent steroid treatment to show similar success to combination therapy giving a median survival time of 570 (2–3540) days and 602 (45–654) days (20, 22). With “non-responders” receiving additional immunosuppressive medications (20). The authors will routinely start a secondary immunosuppressive medication after diagnosis. The two most commonly reported second agent medications (and used by the authors) are Cytosar^®^/ cytosine arabinoside (CA) and cyclosporine (CyA) (1–3, 18, 19, 23–29). The main determining factor between the two, for the authors, is the owner’s ability to transport their pets routinely to a clinic capable of administering chemotherapy. If a hospital is not available nearby or the owners are unable to accommodate appointments at the necessary frequency, cyclosporine is the medication of choice. However, cyclosporine will still require trips to a veterinary hospital capable of venipuncture for medication monitoring, especially at the start of therapy.

#### Cytosine arabinoside therapy

Cytosine arabinoside (cytarabine/ Cytosar^®^/ CA) is useful in the treatment of MUO due to it’s good penetration through the blood brain barrier. Cytosar is an antimetabolite analog medication which, after enzymatic activation, competes with deoxycytidine on DNA resulting in stoppage of DNA replication during the S phase of cellular reproduction. Due to its cell cycle specificity, the duration of cell exposure is important to its efficacy (30). Elimination is primarily via an inactive form through the kidneys (30).

The two clinically reported dosing routes are 200 mg/m^2^ total dose administered as four 50 mg/m^2^ SQ injections given every 12 h or as a CRI (total dose 100–200 mg/m^2^) over 8–24 h (18, 19, 23, 24, 31, 32). Subcutaneous injections should be diluted 2:1 with normal saline (23) and the continuous rate infusion diluted to provide a maintenance fluid rate for the duration of treatment with normal saline.

Subcutaneous injections have failed to reach steady state while it is achieved by hour four of an eight-hour infusion, however, the area under the curve (AUC) is significantly greater over the course of four subcutaneous injections 12 h apart compared to an 8 h CRI (33).

In the treatment of MUO, a significantly larger percentage of dogs initially treated with a constant rate infusion (100 mg/m^2^ over 24 h) lived to 3 months compared to a group receiving subcutaneous injections (50 mg/m^2^ q12h × 4 doses) as the initial treatment (24). Furthermore, a recent study failed to demonstrate an advantage to continued SQ treatments after the initial treatment with CRI (100 mg/m^2^ over 24 h) in dogs with MUO (32). However, these authors choose to continue treatment after the initial CRI administration as demonstrated in previous studies (6, 18, 19, 23, 24, 31). To reduce the number of visits/ lengths of hospitalization for patients (and their owners) studies have compared the pharmacokinetics of different subcutaneous dosing protocols. When comparing the standard 50 mg/m^2^ every 12 h for 4 doses to a single 200 mg/m^2^ dose or 100 mg/m^2^ every 12 h for two doses, it was found that the maximum concentration reached in the plasma was dose dependent but drug exposure measured by area under the curve was similar amongst all dosing protocols (25). Another study compared a 200 mg/m^2^ CRI administered over 24 h to 50 mg/m^2^ administered SQ every 2 h for 4 doses and found the SQ protocol provided higher peak concentrations and sustained plasma levels above its therapeutic target for a significantly longer duration of time than the CRI protocol (31). Once studied more extensively in clinical patients, these alternative treatment protocols could minimize hospitalization for these patients and subsequent client costs along with providing a survival benefit for patients with MUO (25, 31). Ultimately, treatment is continued, via either route, every 3 weeks for three to five treatment cycles. The time between treatments is extended by 1 week each three-to-five-week treatment cycle (18). For example, if every 5 treatments, the treatment interval is extended by 1 week, at the end of year one, the patient would be receiving Cytosar^®^ every 5 weeks and at the end of year two, they would be starting every 7-week treatments. The interval is extended until an every eight-week dosing interval is reached then continued lifelong or discontinued based on patients’ clinical signs and owner preference after consultation with their veterinarian on the risk of relapse if therapy is withdrawn.

Reported median survival times in patients with MUO treated with corticosteroids and Cytosar^®^ are 531 (46–1025) days (23), 78- > 603 days (19), and 26 (0–2250) days (18) with median survival time in dogs surviving 3 months reaching 1616 (562–2241) days (18).

Side effects are possible at higher doses and include bone marrow suppression and gastrointestinal upset. When used an anti-neoplastic agent in dogs, a dose of 300 (100–450) mg/m^2^ administered over 8–72 h resulted in mild to moderate gastrointestinal and bone marrow side effects, but did not require hospitalization to treat (34). However, at the doses commonly reported for use in MUO patients, CBC derangements are rarely clinically significant (19, 23–25, 33). Monitoring of complete blood counts (CBC) is recommended 1 week after treatment for the first 6 months of treatment, then can be intermittently checked bi- or tri-annually thereafter (23, 25, 33). The authors will also monitor the CBC prior to each dose out of an abundance of caution. When administered as a CRI there is an increased side effect profile against the bone marrow, so more frequent monitoring maybe necessary with continued CRI dosing protocols (24, 30). Some clinicians have been increasing the CRI dose of cytarabine to 300 mg/m^2^, but as intermediate to high doses of Cytosar^®^ are used, the potential risk of drug-induced infiltrative lung disease increases. A single case report exists of this side effect in a dog when administered 300 mg/m^2^ dosing (35).

#### Cyclosporine therapy

Cyclosporine targets T-cells through formation of a complex with cyclophilin, with the resultant complex inhibiting calcineurin, a calcium-dependent serine/ threonine phosphatase enzyme. Without calcineurin, the T-cells are unable to produce cytokines such as IL-2, IL-4, IFN-y and TNF-alpha; without these cytokines there is no proliferation of activated T-lymphocytes resulting in suppression of cell-mediated immunity with little impact on humoral immunity. Ultramicronized or microemulsified cyclosporine preparations are effective in dogs, with^1^ Atopica^®^ the only approved medication, however, oral bioavailability is highly variable with further intra- and inter-individual variations present with generic formulations (along with contributions from gastric contents affecting transit times, liver function and concurrent medications affecting the cytochrome P450 pathway) necessitating some form of drug monitoring. Shedding, gingival hyperplasia, anorexia, vomiting and diarrhea are complications; however, vomiting can be reduced by freezing the tablets prior to administration (36). The most clinically significant adverse effect is excessive immunosuppression manifesting as secondary bacterial and fungal bladder, skin and respiratory infections (36–38).

Currently, the only commercially available form of cyclosporine monitoring is pharmacokinetic whole blood levels. Whole blood monitoring is performed on peak (2 h after a dose) and trough (right before a dose) samples. The aim is a peak of 800–1400 ng/ml and a trough of 400–600 ng/ml. However, a variable half-life (ranging from 7 to 10 h, with outliers measured from 1 to 2 h to over 150 h) results in disunity between peak and trough measurements (36, 39). At ideal peak levels, trough levels may be too low and at ideal trough levels, the peak level may be too high (36). In addition, whole blood concentrations and clinical responses are poorly correlated in atopic dogs (39) and levels in the therapeutic range have been found to be clinically effective in some dogs and clinically ineffective in others undergoing immunosuppressive therapy (36).

An alternative to pharmacokinetic testing, is pharmacodynamic testing (only available experimentally). After validation in human studies of the superiority of pharmacodynamic to pharmacokinetic testing, pharmacodynamic testing was developed for dogs measuring the percent suppression of IL-2 expression in cyclosporine treated canine patients. High doses of cyclosporine (10 mg/kg twice daily), reliably suppressed IL-2 production in healthy dogs while lower doses (5 mg/kg once daily) produced variable suppression, with some immunosuppressed and some not, despite comparable whole blood cyclosporine levels (40). Additionally pharmacodynamic testing has determined that healthy dogs will recover to pre-treatment T-cell function by day 3 (range 2–4 days) after discontinuation of cyclosporine (41). In an abstract presentation, comparison of baseline IL-2 expression between healthy dogs and those with auto-immune conditions [inflammatory bowel disease (IBD), immune mediated hemolytic anemia (IMHA), and MUO], found dogs affected with auto-immune diseases to have a higher baseline expression of IL-2 compared to healthy controls. Significance was reached for MUO and IBD dogs compared to healthy controls but not for IMHA. There were no significant differences between the three disease states (42). Previous studies found significant suppression of IL-2 expression within 24 h of oral dosing in healthy dogs treated with cyclosporine (43), however, given the significantly higher baseline IL-2 expression found in our MUO patients, it is reasonable to assume a longer period will be required to fully suppress their T-cell function. When pharmacodynamic testing was available, it was the laboratories recommendation to aim for moderate suppression correlating to 50–80% suppression (44). Based on comparisons with pharmacodynamic testing, peak whole blood levels are more highly correlated with immunosuppression than trough levels (45).

Based on available literature, the authors start cyclosporine at 5 mg/kg twice daily and were previously exclusively using %IL-2 suppression for monitoring but have returned to whole blood level, specifically peak levels, and clinical response to determine the need for dose adjustments. Monitoring is performed one to 2 weeks after the start of cyclosporine therapy and then 1 to 2 weeks after dose adjustments until the ideal level of suppression is achieved. If the patient is clinically improved and peak levels are within range, no change is made to dosage. If the patient is clinically improved and peak levels are over range, dose reductions should be considered to avoid over-immunosuppression. If the patient is not clinically improved and peak levels are below range, dose increases are made. If the patient is not clinically improved but peak levels are above recommended levels, then consideration should be given to switching the immunosuppressive medication for the patient. Monitoring should be completed again before starting the taper of prednisone and every 3–6 months thereafter or when other medications affecting the cytochrome P450 pathway are introduced, removed or the dose adjusted. The most common medication MUO dogs will likely encounter which would affect cyclosporine levels is phenobarbital, which will decrease cyclosporine levels (26). If price is an issue, especially in larger dogs, ketoconazole is administered at 5–8 mg/kg once daily. Ketoconazole inhibits cytochrome P450 enzymes resulting in decreased clearance of cyclosporine (higher peak and trough levels and increased half-life) allowing for once daily dosing of cyclosporine while maintaining the same peak effect (26).

Cyclosporine remains an excellent medication to treat MUO with median survival times of 620, 930, and 1095–1345 days in those cases responding to treatment (26–29).

#### Alternative immunosuppressive therapies

While the authors’ mainstay of treatment is prednisone and Cytosar^®^ or prednisone and cyclosporine, this is strictly clinician preference and other secondary immunosuppressive medications have been studied and proven effective in MUO patients. Procarbazine is a monoamine oxidase inhibitor that crosses the blood brain barrier altering DNA, RNA and protein synthesis. It provides marked cytotoxicity in the S and G_2_ cell divisions. It is dosed orally once daily at 25–50 mg/m^2^, however, with only 50 mg capsules commercially available, compounding is often needed for smaller patients (46). Drug interactions can occur with other medications using the cytochrome P450 pathway or medications affecting serotonin metabolism. Myelosuppression can occur and complete blood counts should be monitored weekly for the first month then monthly thereafter. An inactive form is eliminated through the kidneys (47). Another medication, used by the authors when finances are limited, is azathioprine. Azathioprine is a pro-drug converted to its active form via hepatic metabolism. It causes inhibition of DNA synthesis and mitosis via its actions as a purine analog. The side effect profile includes myelosuppression, hepatotoxicity and pancreatitis. Complete blood count should be monitored during treatment initially every 2 weeks for the first 2 months of therapy then extending to every month thereafter. Serum chemistry should be monitored within 4 weeks of starting azathioprine and the dose reduced by 50% if ALT is >5x increased or discontinued if hyperbilirubinemia develops (48). It is dosed orally at 2 mg/kg once daily in small patients and 50 mg/m^2^ in larger patients. After 2 weeks, the dose frequency is reduced to every other day. This would lead, long term, to the ability to dose MUO patients 1 day with prednisone and the next with azathioprine (49). Mycophenolate mofetil is another medication preferred by some neurologists and like Cytosar^®^ (and unlike cyclosporine, procarbazine and azathioprine), it comes as an intravenous formulation for those patients who are unable to take oral medications due to neurologic abnormalities at the start of treatment. It works through inhibition of the *de novo* pathway of purine synthesis to suppress proliferative responses of both B- and T-cell lymphocytes. Unfortunately, up to 20% of patients started on mycophenolate will develop gastrointestinal adverse effects (hemorrhagic diarrhea) within the first 2 weeks of treatment which can be dose limiting (50). Mycophenolate is dosed at 10–20 mg/kg twice daily with adverse effects more likely at the higher end of the dose range (51). Other potential adverse effects include myelosuppression and hepatoxicity, necessitating the need for routine complete blood count and serum chemistry assessment throughout the treatment period. Proton pump inhibitors, ciprofloxacin and amoxicillin/ clavulanic acid should not be used in patients treated with mycophenolate as they have been found to reduce the mycophenolate concentrations. A liquid oral formulation exists but will increase the exposure potential when clients are administering to veterinary patients (51).

When tertiary medications are needed, Cytosar^®^ can be added to prednisone and cyclosporine or procarbazine combinations (19, 23, 52). Mycophenolate and cyclosporine have been used concurrently with a reduction in mycophenolate dose (51), however, mycophenolate and azathioprine should not be used together.

#### Other factors

Lifestyle changes should be recommended for MUO patient households. Vaccination of MUO patients remains a controversial subject with studies unable to find a correlation to the disease or recrudesce of disease once in remission, however, the use of live attenuated vaccines should be avoided in patients undergoing immunosuppressive treatment and other vaccinations may be less effective (36). Dogs undergoing immunosuppressive therapy should avoid high traffic areas such as dog parks, doggy daycare and boarding situations where they may be exposed to communicable diseases. Clients who are handling these immunosuppressive medications in the process of oral administration and their pets’ secretions while on these immunosuppressive medications should be properly counseled in safe handling procedures. In addition, they should be counseled on the risks to conception and pregnancy posed with these medications (30, 36, 47, 48, 51).

## Conclusion

The pharmacologic strategy in the treatment of MUO requires immunosuppression +/− anti-epileptic medications in the long-term along with additive supportive care in the short term for elevated intracranial pressure. The most commonly reported immunosuppressive combinations from the literature are steroids in combination with Cytosar^®^ or cyclosporine, which coincides with the authors preferences. However, with the lack of prospective controlled studies in the treatment of MUO, no treatment can be confirmed superior to another. When deciding on treatment of choice, clinicians must use their own experiences and preferences along with the financial and travel abilities of their clients to determine the ideal medication combination for each patient.

## Author contributions

All authors listed have made a substantial, direct, and intellectual contribution to the work and approved it for publication.

## Conflict of interest

The authors declare that the research was conducted in the absence of any commercial or financial relationships that could be construed as a potential conflict of interest.

## Publisher’s note

All claims expressed in this article are solely those of the authors and do not necessarily represent those of their affiliated organizations, or those of the publisher, the editors and the reviewers. Any product that may be evaluated in this article, or claim that may be made by its manufacturer, is not guaranteed or endorsed by the publisher.

## Footnotes

^1^Elanco Animal Health, Greenfield, IN, USA.
